# Ankle joint salvage and reconstruction by limited ORIF combined with an Ilizarov external fixator for complex open tibial pilon fractures (AO 43-C3.3) with segmental bone defects

**DOI:** 10.1186/s12891-022-05060-y

**Published:** 2022-01-28

**Authors:** Yu Chen, Yaxing Li, Xiangyu Ouyang, Hui Zhang

**Affiliations:** 1 Department of Orthopedics, Orthopedic Research Institute, West China Hospital, Sichuan University, No. 37, Guoxue Avenue, Chengdu, 610041 Sichuan Province China; 2 Disaster Medicine Center, Sichuan University, Chengdu, 610041 Sichuan Province China; 3 Department of Orthopedics, Chengdu Office Hospital of Tibet Autonomous Region, branch Hospital of West China Hospital, Sichuan University, Chengdu, 610041 Sichuan Province China

**Keywords:** Pilon, Open fracture, Bone defect, Bone grafting, Ilizarov, ORIF

## Abstract

**Background:**

Open pilon fractures combined with sizeable segmental bone defects are rare, difficult to treat, and often result in the loss of ankle joint function. The purpose of this study was to determine clinical outcomes in patients with open pilon fractures and sizeable segmental bone defects treated by limited ORIF combined with an Ilizarov external fixator.

**Methods:**

We conducted a retrospective analysis of open pilon fractures with sizeable segmental bone defects treated by limited ORIF combined with the Ilizarov external fixator strategy between July 2014 and August 2019. All patients were included for assessments of fracture healing and infection rates. Ankle functional outcomes were assessed in all patients according to the Paley criteria and American Orthopedic Foot and Ankle Society Score (AOFAS) at least 24 months post-injury.

**Results:**

All patients were followed up for a mean of 41.09 months. The mean bone defect size was 5.64 ± 1.21 cm. The average EFI and BTI were 1.56 ± 0.28 months/cm and 11.12 ± 0.74 days/cm, respectively. According to the Paley evaluation system, the success rate of ankle joint reconstruction was 64% (7/11). The mean score based on the AOFAS functional assessment was 77.73 ± 8.87. Five patients showed posttraumatic arthritis, one of whom required ankle arthrodesis. Three patients developed pin site infections, and one patient developed a deep infection after bone grafting.

**Conclusion:**

The strategy of limited ORIF combined with an Ilizarov external fixator can restore ankle function in most patients with complex open tibial pilon fractures. Ankle stiffness, pin tract infection, and traumatic arthritis were the most common complications associated with this therapy.

## Background

Tibial pilon fractures account for 1% of lower limb fractures and approximately 3–10% of tibial fractures [1]. They are generally a result of high-energy mechanisms, such as high-altitude falls and motor vehicle accidents, resulting in an open fracture in approximately 10–30% of cases [2]. According to the AO/OTA classification system, complex tibial pilon fractures (AO 43-c3.3) are one of the most severe types of pilon fractures, including comminuting fractures of the distal articular surface and metaphysis of the tibia, while an open wound poses additional problems, including bone defects [3, 4].

Due to the low incidence of complex open tibial pilon fractures with sizeable segmental bone defects, few reports are currently available, and the treatment is extremely challenging [5]. Although early reconstruction and repair of the articular surface and bone defects constitute an essential prerequisite for restoring ankle joint function, implementing such procedures at the appropriate time is challenging because of the risk of infection due to open soft tissue injury [6, 7]. Previous studies have reported that most patients with pilon fractures and large bone defects undergo ankle arthrodesis [3, 8]. We attempted limited open reduction combined with an Ilizarov external fixation in such patients to restore ankle function. The purpose of this study was to determine clinical outcomes in patients with open pilon fractures and sizeable segmental bone defects treated with limited ORIF (open reduction and internal fixation) combined with an Ilizarov external fixator.

## Methods

With approval from the Ethics Committee on Human Research of our hospital (NO.2021–210), the surgical database was reviewed for all pilon fractures treated over a consecutive 5-year period at a major trauma center in western China. A total of 834 pilon fracture cases were treated at the Department of Orthopedics, West China Hospital, Sichuan University (Sichuan, China) from July 2014 to August 2019. Among them, 142 patients with C3 pilon fractures, including 77 patients with open fractures, were selected strictly according to the inclusion criteria, and 11 patients were finally included to form the study group (Table 1).

**Table 1:** Patient Data

case	Age-range (yrs.)	Mechanism	Side	AO/OTA Classification	Gustilo Type	Smoker?	Diabetes?
1	30–35	MVA	L	43C3.3	IIIA	N	N
2	40–45	Crush	L	43C3.3	IIIB	Y	N
3	40–45	Fall	R	43C3.3	IIIA	N	N
4	50–55	Fall	L	43C3.3	IIIA	Y	N
5	45–50	MVA	L	43C3.3	IIIA	Y	N
6	30–35	Fall	R	43C3.3	IIIA	N	N
7	40–45	Fall	L	43C3.3	IIIA	N	N
8	55–60	MVA	L	43C3.3	IIIB	Y	N
9	25–30	Crush	R	43C3.3	IIIB	N	N
10	30–35	MVA	R	43C3.3	IIIA	N	N
11	35–40	Fall	R	43C3.2	IIIA	N	N

*F* Female, *M* male, *L* left, *R* right, *Y* yes, *N* no, *MVA* motor vehicle accident

The inclusion criteria were as follows: (1) AO/OTA C3 open pilon fracture with a metaphyseal bone defect ≥4 cm (a sizeable bone defect was defined as a bone defect ≥4 cm in this study); (2) application of the bone transport technique and limited ORIF; and (3) follow-up for more than 1 year, with complete follow-up data. The exclusion criteria were (1) confirmed infection at admission; (2) loss to follow-up; (3) preoperative examination revealing limited ankle function due to nerve injury; and (4) potential limb ischemia risk due to vascular injury.

Charts and radiographs were reviewed retrospectively to determine patient demographics and comorbidities, injury mechanisms, open fracture classifications according to Gustilo and Anderson [9], AO/OTA fracture classifications [10], the clinical course, the time to healing, and complications. The patients included seven males and four females with an average age of 40.55 ± 9.43 years (29–58 years). Among the 11 patients, 4 were smokers without diabetes (Table 1**)**.

### Treatment strategies

**Fig. 1:**
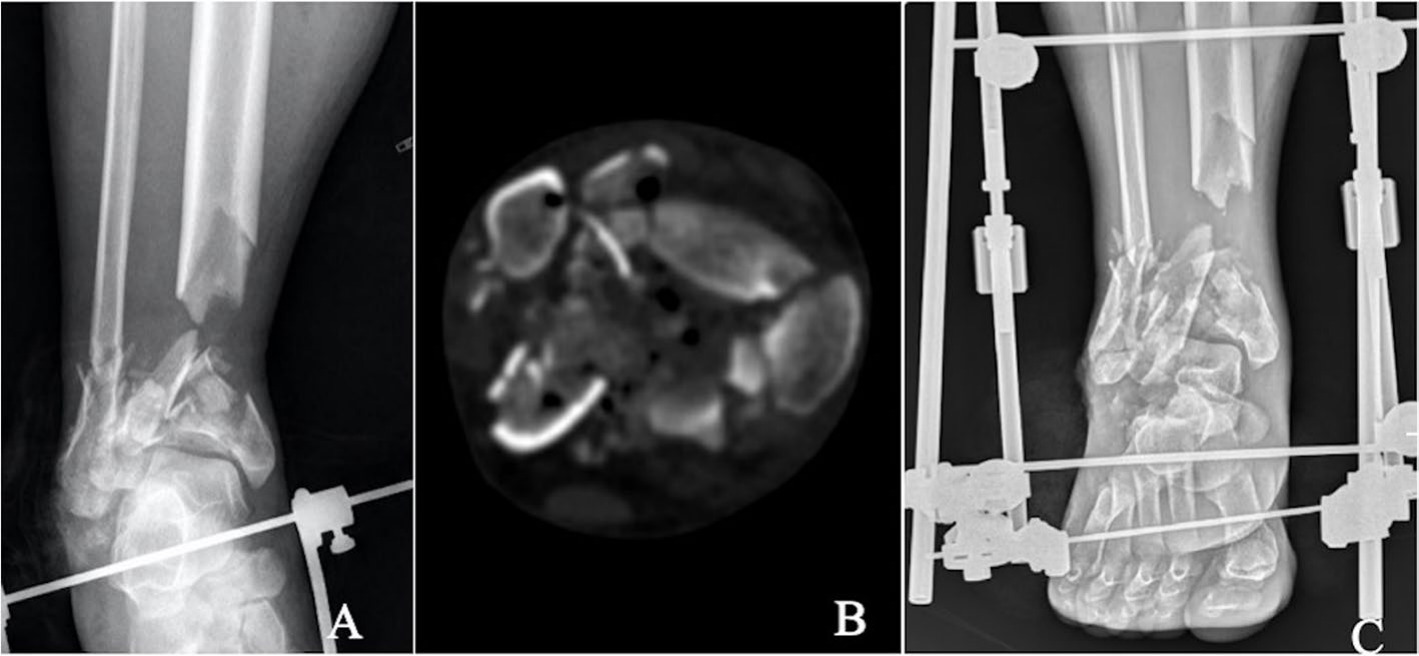
Anteroposterior (**A**), and computed tomography (CT) scan (**B**) of a middle-aged patient who fell from a height and sustained a type IIIA open AO/OTA type C3.3 pilon fracture among other injuries. Anteroposterior (**C**),immediate debridement, spanning external fixation. The distal tibial metaphyseal defect was evident

**Fig. 2:**
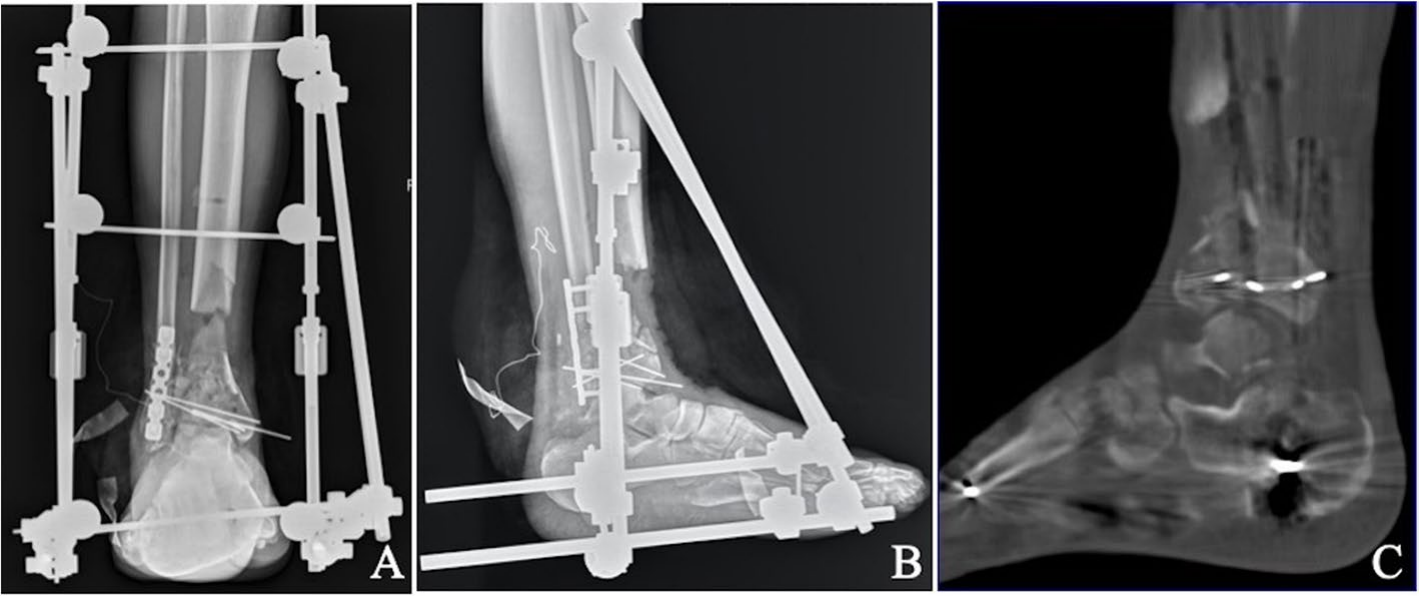
Anteroposterior (**A** & **B**) X -ray:after 15 days, thorough debridement and bone resection were done. The resulting defect was 6 cm in length. Limited ORIF was performed with K wires and fibular plate fixation was performed. Computed tomography (CT) scan (**B**): shows joint reduction, step or gap < 2 mm

Treatment strategies were carried out in stages. The primary purpose of the first stage was to transform the open contaminated wound into a clean wound and stabilize the ankle joint. The main measures included radical debridement, reduction and limited fixation of the distal tibial articular surface, transmalleolar triangular external fixator application, and the use of a VAC negative pressure covering for the wound (Fig. 1). The first stage requires radical debridement, complete removal of contaminated free epiphyseal fractures, and soft tissue deactivation using a traumatic incision or an appropriate independent surgical approach. Any fracture fragments with intact soft tissue attachments and fracture fragments of all joints should be retained. Articular surface reconstruction was then completed by reducing the fracture fragments, followed by fixation with screws or Kirschner wires (Fig. 2). At the same time, blood vessels and nerves were evaluated to determine whether a blood supply disorder was present and the possibility of denervation. Patients were then informed of the severity of the injury, the risks, and possible complications.

Articular reconstruction was not performed during the initial debridement surgery for several reasons, such as severe wound contamination or a long preoperative waiting time. At the same time, a simple triangular external fixator was used for joint distraction and fixation to achieve initial ankle joint stability. Postoperative three-dimensional computed tomography (CT) examination can better depict the displacement of the distal tibia articular surface and facilitate better preoperative planning for the next step of the articular surface reduction plan. During early debridement, antibiotic bone cement beads loaded with vancomycin and gentamicin were placed at the bone defect or wound surface. The VAC (KCI, San Antonio, TX) covered skin and soft tissue defect wounds.

Blood inflammatory indices, including white blood cells, the erythrocyte sedimentation rate (ESR), and C-reactive protein (CRP), were tested at 1, 3, 5, and 7 days postoperatively. Delayed open reduction and limited internal fixation of the distal tibia were performed when the inflammatory index was lower than two times the standard. Bone cement beads loaded with the antibiotics vancomycin and gentamicin were also placed at the defect. Open reduction of the fracture can be performed by an anterolateral, posterolateral, or combined approach outside the nontraumatic incision. Most patients with fibular fractures were treated with open reduction and plate fixation through a posterolateral approach during the initial surgery. Small microfracture fragments were fixed with Kirschner wire during joint reconstruction, while larger bone fragments were fixed with screws.

**Fig. 3:**
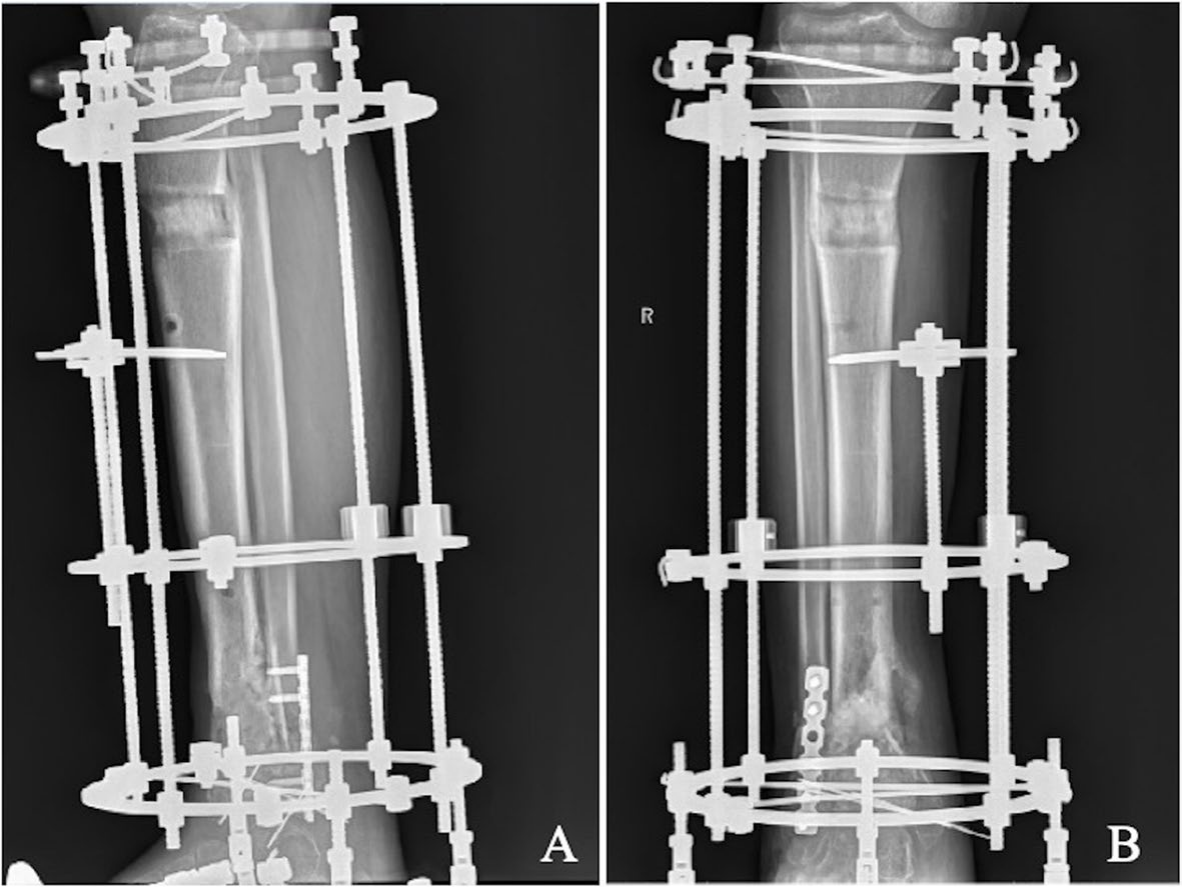
Gradual bone transport was performed (**A**, **B**). At the end of bone transport, autologous iliac bone grafting is used at the bone defect in the original fracture area of the distal tibia

The goal of the second stage was the treatment of bone defects and firm fixation for early functional exercise. The main measures were tibial Ilizarov external fixator fixation, bone transport, and autologous bone grafting (Fig. 3). An Ilizarov circular frame external fixator (Beijing Institute of Exoskeleton Fixation Technology, China) was used for tibial external fixation. Two fine wires and a half pin were placed at the proximal and distal ends of the tibial fractures. Then, foot components were assembled on the external bracket to fix the tibia and foot simultaneously, and hinges were installed on the ankle joint plane to ensure that the ankle joint was movable. At the same time, the Ilizarov bone transport technique was used to fill the bone defect through bone transport, and the osteotomy site was selected 1 cm below the tibial tuberosity near the tibia. After 1 week, the distal tibia was gradually moved at a rate of 1 mm/day. X-ray examination was performed 2 weeks after surgery to ensure the correct movement direction and distance of the osteotomy until the gap between the two osteotomy ends was less than 1 cm. When bone transport was complete, the antibiotic bone cement beads were removed. Cancellous bone is obtained from the iliac bone as a bone graft. The scar and soft tissue of the original bone defect of the distal tibia were cleaned, and the cancellous bone from the ilium was transplanted to the bone defect of the distal tibia. Bone grafts were applied 8–10 weeks (range, 5–20 weeks) after joint reconstruction on average.

### Assessments

Plain radiographs, CT scans, and full-length lower extremity radiographs were obtained to assess the quality of consolidation, bone union, the mechanical axis and the length of the lower extremity. The external fixation time (EFT) was recorded from Ilizarov external frame installation to fixator removal. The external fixation index (EFI) was calculated as the EFT divided by the traction length. The bone transport time (BTT) refers to the duration of bone transport; similarly, the bone transport index (BTI) refers to the bone transport time divided by the length of bone transport. Joint spacing and clearance were measured as the distance between fracture fragments of the tibial articular surface, where articular malreduction was defined as joint steps or clearance ≥2 mm. The bone and functional results were assessed by the Paley criteria [11]. In this study, the success of ankle reconstruction was defined as both bone and function scores of “good” or “excellent” according to Paley criteria. The patients were instructed to report their exercise capacity: walking, running, jumping, squatting, and traveling up/down stairs. The response choices were “easy,” “slightly difficult,” “difficult,” and “unable” [12]. All complications and sequelae were recorded.

## Results

**Fig. 4:**
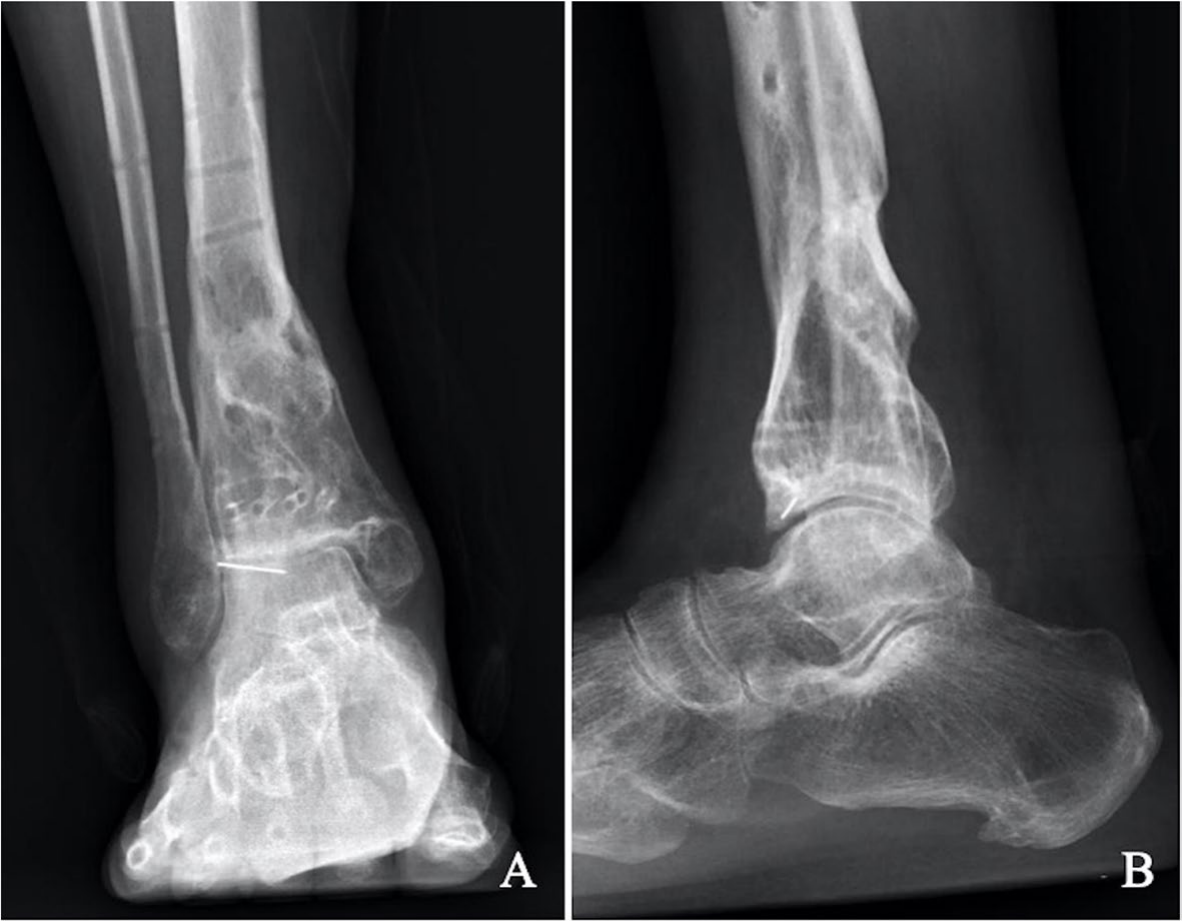
At the 20-month follow-up, plain radiographs show bone healing and good alignment (**A**, **B**). He had returned to normal activities with complaints of mild intermittent ankle pain and stiffness

The mean radiographic and clinical follow-up for all patients was 41.09 months (range 25–75 months). The mean bone defect size was 5.64 ± 1.21 cm (range 4–7 cm). The external fixator was removed after fracture union was confirmed by imaging examination during the follow-up (Fig. 4). The mean external fixator time was 8.82 ± 2.56 months (range 6–14 months). The mean bone transport time was 63.09 ± 16.1 days (range 40–90 days). The external fixator index—the number of months that the patients wore the fixator for each additional centimeter—averaged 1.56 ± 0.28 months/cm (range 1.33–2.33 months/cm). The bone transport index was 11.12 ± 0.74 days/cm (range 10.00–12.86 days/cm). Patients underwent an average of 4.27 ± 1.27 surgeries (range 3–7) during the entire treatment period **(**Table 2).

**Table 2:** Clinical Data

case	Follow-up (month)	Days Between Stages 1 and 2 (day)	Size of Bone Defect (cm)	Graft Used	operation frequency^a^	EFT (month)	BTT (day)	BTI (day/cm)	EFI (month/cm)
1	32	12	4	Autograft	3	6	45	11.25	1.50
2	75	7	7	Autograft,	5	10	75	10.71	1.43
3	36	15	6	Autograft,	4	8	67	11.17	1.33
4	28	17	6	Autograft	3	9	65	10.83	1.50
5	33	11	5	Autograft	5	8	55	11.00	1.60
6	38	14	7	Autograft	5	10	80	11.43	1.43
7	25	13	4	Autograft	3	6	40	10.00	1.50
8	41	8	4	Autograft	5	6	42	10.50	1.50
9	29	9	6	Autograft	3	8	70	11.67	1.33
10	52	7	6	Autograft	4	14	65	10.83	2.33
11	63	16	7	Autograft	7	12	90	12.86	1.71

^a^ Procedures include the usual debridement procedures, internal fixation, external fixation, soft tissue treatment, and bone grafting, but do not include external fixator removal after healing

*EFT* external fixator time, *BTT* bone transport time, *BTI* bone transport index, *EFI* external fixator index

The articular surface reduction in 11 patients was excellent without malunion based on imaging evaluations. At the final follow-up, according to the Paley criteria, the bone result was classified as “excellent” in ten patients and “poor” in one patient, while the functional result was graded as “good” in seven patients and “fair” in four patients. The success rate of ankle joint reconstruction was 64% according to the Paley evaluation system (7/11). According to the AOFAS ankle-hind foot function evaluation, the average score was 77.73 ± 8.87 (range, 65 to 87). The range of motion (ROM) of the affected ankle at the last follow-up examination was excellent in four patients (at least 35°), good in three patients (20°-35°), fair in three patients (0°-20°), and poor in one patient (0°). All patients were independently ambulant at the final review; seven returned to their original employment, two returned to lighter duties, and two were unemployed before their injuries **(**Table 3**)**.

**Table 3:** Clinical outcomes

Case	Gap/step of articular surface (mm)	Paley criteria		AOFAS	Range of motion (ROM)			Exercise capacity				
		bone result	functional result		Dorsiflexion (°)	plantarflexion (°)	Range (°)	Walking	Running	Jumping	Squatting	Going up/down the stairs
1	<2	Excellent	Good	87	30	5	35	Easy	Easy	Easy	Easy	Easy
2	<2	Excellent	fair	67	10	0	10	Easy	Little difficult	Easy	difficult	Little difficult
3	<2	Excellent	fair	68	10	0	10	Easy	Easy	Easy	Easy	Little difficult
4	<2	Excellent	Good	79	25	5	30	Easy	Easy	Easy	Easy	Easy
5	<2	Excellent	Good	78	15	10	25	Easy	Easy	Easy	Easy	Easy
6	<2	Excellent	Good	89	30	10	40	Easy	Easy	Easy	Easy	Easy
7	<2	Excellent	fair	69	15	0	15	Easy	Easy	Little difficult	Little difficult	Little difficult
8	<2	Excellent	Good	92	30	5	35	Easy	Easy	Easy	Easy	Easy
9	<2	Excellent	Good	87	25	5	30	Easy	Easy	Little difficult	Easy	Easy
10	<2	Excellent	Good	86	30	5	35	Easy	Easy	Easy	Easy	Easy
11	<2	Poor	fair	65	0	0	0	Easy	difficult	Unable	difficult	difficult

Five patients showed evidence of posttraumatic arthritis, one of whom required ankle arthrodesis (case 2). Elongated callus curvature was observed in one patient (Case 10) after removal of the fixator, which must be corrected in a subsequent surgery. Needle infection occurred in 3 patients, two of whom were cured after dressing changes, and the other patient’s condition improved after reconstruction and replacement of fixation needles. Their results were considered good. Functionally, six patients complained of ankle stiffness due to the extension of the external fixator into the foot, which improved with satisfactory results after 4 months of physical therapy **(**Table 4**)**.

**Table 4:** Complications

Case	Complications	Treatment measures	outcome
1	Superficial wound infection; Needle infection	dressing change	Infection cure
2	OA; Needle infection; ankle stiffness	required ankle arthrodesis dressing change; Rehabilitation training	Infection cure; Recovery of joint range of motion
3	OA; ankle stiffness	Rehabilitation training	Recovery of joint range of motion
4	OA; ankle stiffness	Rehabilitation training	Recovery of joint range of motion
5	OA; ankle stiffness	Rehabilitation training	Recovery of joint range of motion
6	NO	/	/
7	OA; ankle stiffness	Rehabilitation training	Recovery of joint range of motion
8	Superficial wound infection; Needle infection; OA	replacement of fixation needles.	Infection cure
9	ankle stiffness	Rehabilitation training	Recovery of joint range of motion
10	Elongated callus curvature	This needs to be corrected in a subsequent surgery	Limb length and force line correction
11	Deep wound infection; ankle stiffness	the infected distal tibia was removed and tibial distance fusion was performed after infection control.	Loss of ankle function

One patient (case 11) underwent seven irrigation and debridement procedures, and the case was considered a failure. After iliac autografting was performed in this patient nine weeks postoperatively, the wound was infected, and *Staphylococcus aureus* and *Enterococcus faecalis* were cultured. Infection control was not reasonable after three expansion surgeries. Finally, the infected distal tibia was resected, and ankle joint arthrodesis was performed after infection control.

## Discussion

Complex open tibial pilon fractures (AO 43-C3.3) with sizeable segmental bone defects are associated with high-energy mechanisms and significant soft tissue injury by definition and represent a rare type of severe pilon fracture. The incidence rates in the trauma center (Level I) of our hospital are 1.3% (11/834) among all pilon fractures and 14.3% (11/77) of C3-type open pilon fractures. To date, only a few case reports [1, 7, 8] have described successful treatment experiences for this type of serious injury. This type of injury is rare, and treatment experiences are not extensive and systematic, which renders these injuries challenging to manage, with little published literature available to guide surgeons. This article introduces and analyzes a case series of C3-type open pilon fracture treatment with large bone defects using a systemic treatment plan (staged treatment strategy: limited ORIF technology to reconstruct the tibial articular surface combined with an Ilizarov circular frame external fixator and bone transportation technology to treat bone defects).

The goal of treatment of complex open tibial pilon fractures with sizeable segmental bone defects (AO 43-C3.3) is to avoid infection and soft tissue necrosis while achieving articular surface reconstruction and bone defect healing, which may be challenging [13]. While operations over several stages are currently a popular treatment method considering soft tissue injuries [14, 15], recently, some articles have recommended early soft tissue covering of the open wound using a vascularized muscle flap [16, 17]. However, in these cases, articular surface reconstruction and bone defect healing are performed after the wound has healed, which leads to severe articular cartilage degeneration and difficulty in anatomical reduction of the joint. Recent studies have reported that initial arthrodesis has an excellent functional prognosis [18–21]. However, the literature also mentions that ankle fusion increases the risk of arthritis in adjacent joints, especially when the subtalar joint and the foot are involved [13, 22]. In addition, initial arthrodesis for pilon fractures with bone defects may cause shortening of the limbs.

Regardless of bone defects, surgeons have adopted various strategies to address the challenge of open pilon fractures. Nevertheless, regardless of which treatment is used, the results of studies have shown that these injuries are severe and lead to long-term dysfunction [23]. For bone defects larger than 4 cm in the metaphysis, bone transport technology replaces conventional bone graft surgery (cancellous bone autograft, structural bone allograft, demineralized bone matrix, and calcium-based cement) to solve this problem well. Early open reduction and internal fixation (ORIF) have been reported to be associated with severe soft tissue complications and deep wound infection for open pilon fractures [24]. Compared with plate fixation, external fixation for open pilon fractures has also been reported to reduce the incidence of complications but to increase the variability of reduction of tibial epiphyseal and articular surface fractures [23]. Therefore, in recent years, an increasing number of surgeons have tended to adopt limited internal fixation combined with external fixation to limit irritation to skin and soft tissue [25, 26].

The method that we used, limited ORIF joint reconstruction combined with Ilizarov bone transport technology to treat bone defects, can better preserve the ankle joint range of motion, and the incidence of soft tissue complications is low. The advantages of the method are that the joint can be reduced and fixed early, which reduces the difficulty of joint reduction and slows articular cartilage degeneration. Second, it also provides sufficient strength to allow patients to bear weight and engage in functional exercises early, and the technology of external fixator fixation combined with bone grafting can effectively treat sizeable bone defects larger than 4 cm, thus avoiding limb shortening. At the same time, in bone transportation, skin stretching can be performed to solve the problem of soft tissue coverage [27, 28]. On the whole, the strategy of multi-stage treatment plays an essential role in the rescue of ankle joint function. Early joint reconstruction in the initial stage can significantly reduce the chance of infection caused by open soft tissue injury; the Ilizarov external fixator used in the second stage can avoid bone non-union caused by the bone defect and provide conditions for early rehabilitation of the ankle joint.

Although the clinical results appear to be satisfactory, we also observed complications. The most common issue is that the external fixator is worn for a long time, and a close follow-up is required to monitor bone ingrowth, joint movement, and needle tract infection, loosening, or breakage. To prevent needle tract infection, the patients were instructed to pay attention to personal hygiene, disinfect the needle tract with iodophor twice a week and cover the tract with a sterile dressing. Our study also described three patients with needle tract infections, 2 of whom were successfully treated with needle injection or local injection of antibiotics, and 1 patient was treated with half-needle reinsertion.

However, the proximity of the injury to the joint often mandates that the joint be spanned. Fixation of a fixed joint increases the likelihood of tibiotalar or subtalar joint stiffness or both [29, 30]. Six patients complained of a stiff ankle because of the need to extend the frame down to the foot, which was improved by 4 months of physiotherapy with satisfactory results. Physiotherapy is vital during and after bone transport until the frame is removed and the patient returns to regular activity [31]. According to the AOFAS ankle-hind foot function evaluation, the average score was 77.73 (range, 65 to 87), which was excellent and good in seven patients.

The optimal long-term outcome following an open pilon fracture includes avoidance of soft tissue complications and osseous anatomy restoration [16]. Restoration of limb alignment and rotation, along with anatomic restoration of the joint surface, is imperative, as the development of posttraumatic arthritis has been shown to correlate closely to the severity of the injury and the quality of reduction [32]. In this study, all patients had excellent reduction of the articular surface early, and no manifestations of infection were noted. In theory, postoperative ankle degeneration should not occur, but in actual follow-ups, nearly 50% (5/11) of patients were found to have joint degeneration on imaging, and one patient had clinical symptoms. Thus, further effort is still required with respect to restoring ankle joint function after this injury. We should also focus on the long-term effects of osteochondral, ligament, joint stability, and other aspects on the function of the ankle joint.

After bone transport is completed, the patient must undergo a secondary bone graft at the docking site to promote bone healing of the broken end. Although previous recommendations regarding the timing of bone grafting for open pilon fractures vary, acute bone grafting is contraindicated in open fractures with contaminated wounds [2,17,34]. Even when the wound is initially closed and no sign of infection is evident after bone metastasis, subclinical contamination is still a problem, and we think that using antibiotic-impregnated beads to thoroughly disinfect the wound before the final bone graft is wise. Although we used antibiotic bone cement beads, one patient had a delayed infection after bone grafting. We should be extra careful when bone grafting and recommend using autologous cancellous tissue premixed with vancomycin when the bone is grafted.

Certain limitations are evident in the current study. First, this is a retrospective study with a limited sample size. Second, no control group was included for comparison. Based on the literature survey and clinical experience, we believe that patients with bone defects greater than 4 cm are suitable for bone transport. Notably, although this new strategy was successful in 90% of patients deemed suitable for bone transport and articular reconstruction, the study sample was a carefully selected group. This technique was not applicable to many other patients with acute deep infection, poor soft tissues, or other physiological factors. However, we will strictly follow the treatment protocol once a patient is judged to be suitable for the strategy.

## Conclusion

The new therapeutic strategy, where sizeable segmental tibial bone defects are managed using the Ilizarov technique and reconstruction of the tibial plafond by limited ORIF, provides additional options for surgeons when managing open pilon fractures with sizeable bone defects. Ankle stiffness, pin tract infection, and traumatic arthritis were the most common complications associated with this therapy. Proper patient selection and management are the keys to the success of this technique. However, prospective studies with larger sample sizes are required to confirm our findings.

## Data Availability

Data are available from West China Hospital, China. The datasets used and/or analyzed during the current study are available from the corresponding author on reasonable request.

## Abbreviations

ORIF: Open Reduction with Internal Fixation
AOFAS: American Orthopedic Foot and Ankle Society Score
EFT: External fixation time
EFI: External fixation index
BTT: Bone transport time
BTI: Bone transport index
CT: Computed tomography
ESR: Erythrocyte sedimentation rate
CRP: C-reactive protein
ROM: The range of motion
OA: Osteoarthritis
